# Acknowledgement to Reviewers of *Proteomes* in 2014

**DOI:** 10.3390/proteomes3010001

**Published:** 2015-01-08

**Authors:** Proteomes Editorial Office

**Affiliations:** MDPI AG, Klybeckstrasse 64, CH-4057 Basel, Switzerland

The editors of *Proteomes* would like to express their sincere gratitude to the following reviewers for assessing manuscripts in 2014:

Agrawal, Ganesh
Albrecht, Huguette
Atkinson, Michael
Auais, M.
Azimzadeh, Omid
Baulch, Janet E.
Benedetti, Celso Eduardo
Briza, Peter
Brown, Celeste J
Brummer, Tilman
Carpentier, Dr. Sebastien
Casati, Paula
Castro, Antonio Jesus
Cheema, Amrita K.
Chen, Sixue
Cione, Erika
Cristobal, Susana
Cui, Jiuwei
Dam, Svend
Fornace, Albert J.
Galván, José Valero
Garbett, Nichola C.
Ghosh, Dipanjana
Gosti, Françoise
Greinert, Rüdiger
Hajduch, Martin
Hartl, Markus
Heazlewood, Josh
Hu, Jianping
Jander, Georg
Juranek, Judyta Karolina
Kletsas, Dimitris
Kosová, Klára
Lan, Ping
Li, Ka Wan
Lu, Mingyang
Lukkahatai, Nada
Luthe, Dawn
Mann, Karlheinz
Margaritis, Lukas H.
Martinez, Inigo
Messori, Luigi
Metzger, Jochen
Mundy, John
Murray, Graeme I.
Oliveira, J.T.A.
Pancsa, Rita
Pontarotti, Pierre
Quong, Andrew A.
Rödel, Franz
Rodriguez-Celma, Jorge
Ruggieri, Rosamaria
Santini, Valeria
Sava, Gianni
Schiller, Martin R.
Sgarra, Riccardo
Sheehan, David
Shen, Wenbiao
Shrivastava, Indira
Siede, Wolfram
Simova-Stoilova, L.
Smalla, Karl-Heinz
Šuštar-Vozlič, Jelka
Terenzi, Hernán F.
Trivedi, Arun K.
Unger, Elizabeth R.
Unger, Ron
Uversky, Vladimir N.
Wakeel, Abdul
Wienkoop, Stefanie
Woo, Sun-Hee
Woriedh, Mayada
Yang, Austin J.

